# “We know that our voices are valued, and that people are actually going to listen”: co-producing an evaluation of a young people’s research advisory group

**DOI:** 10.1186/s40900-023-00419-4

**Published:** 2023-03-20

**Authors:** Louca-Mai Brady, Jacqueline Miller, Eleri McFarlane-Rose, Jasmine Noor, Rhianne Noor, Annegret Dahlmann-Noor

**Affiliations:** 1 Richard Desmond Children’s Eye Hospital, Moorfields Eye Hospital, 3 Peerless Street, London, EC1V 9EZ UK; 2 Present Address: Centre for Research in Public Health and Community Care School of Health and Social Work, University of Hertfordshire, Hatfield, AL10 9AB UK

**Keywords:** Public involvement, PPI, Co-production, Young people, Paediatric research, Evaluation, Eye and vision research, Ophthalmology

## Abstract

**Background:**

Children and young people’s (CYP) involvement is an increasing priority in UK healthcare and in heath research, alongside recognition that involving CYP in research requires different considerations to involving adults. Underpinned by children’s rights and a co-production ethos this paper, co-authored with young evaluators, explores the learning from a co-produced evaluation of eyeYPAG, a young persons’ research advisory group (YPAG) for eye and vision research based at Moorfields Eye Hospital, London, UK.

**Methods:**

A team of young evaluators, supported by the eyeYPAG facilitator, conducted focus groups and online surveys with YPAG members, their parents and carers, researchers, group facilitators and funders. Qualitative data was analysed using a collaborative reflexive thematic analysis approach. Quantitative data, limited by the small number of participants, was analysed in Excel and reported as descriptive data.

**Results:**

CYP valued the social and creative aspects of the group as well as learning about research and developing skills and confidence. Learning was a two-way process, with both researchers and facilitators reflecting on how much they had learnt from working with the YPAG. All participants talked about the importance of impact, feeling that CYP are making a difference to research, as well as CYP’s right to be involved. Effective planning and facilitation were key to the success of the group, in relation to accessibility and the development and delivery of sessions both online and in-person. Resourcing and administration were key challenges to this, as was engaging researchers who were not already converted to the public involvement cause. As the nature of a YPAG is that it primarily focuses on advising researcher-led projects, co-production was identified as something that the group was ‘working towards’, including through this evaluation. Co-producing with CYP involves building up knowledge, confidence and acknowledging power dynamics.

**Conclusions:**

Co-producing an evaluation enabled us to learn about the benefits and challenges of involving CYP in research, as well as how to involve them in the development of that evidence. An ethos of co-production and children’s rights helped to shift the balance of power and develop more engaging and inclusive ways of working.

## Abstract (summary)

Children and young people (CYP) have a right to be involved in things that affect them, including research. There is growing interest in children and young people’s involvement in health research in the United Kingdom (UK), as well as understanding that what works for CYP is often different to what works for adults. This paper presents an evaluation of the Young Person's Advisory Group (YPAG) at Moorfields Eye Hospital in London. Evaluation uses research methods to find out how well a service or project is working and meeting the needs of those who use it, and how to improve things that could be better. A group of young evaluators, supported by adult researchers, designed and ran the evaluation; three of the young evaluators also helped write this paper. In our evaluation we used focus (discussion) groups and online surveys with young group members, their parents and carers, researchers who had worked with the group, the group’s facilitators (adults who help manage the group) and funders. We found that group members valued the social and creative aspects of the group as well as learning about research and developing skills and confidence. Learning was a two-way process, with both researchers and facilitators talking about how much they had learnt from working with the YPAG. All participants talked about the importance of feeling that CYP are making a difference to research, as well as of CYP’s right to be involved. Planning and support were important to the group working well, but we found that having the money and time to do this well was not always easy. And, while lots of researchers were keen to work with the group, and talked about how this had helped their research, we need to do more to engage researchers who have yet to be convinced. We also found that, while we wanted to ‘co-produce’ the group and share power for all big decisions, this was something we had to work towards, especially when group members were young and/or new to research and involvement. Co-producing an evaluation helped us to learn about the benefits and challenges of involving CYP in research, as well as how to involve them in evaluating that involvement.

## Background

### Involving children and young people in research

Involving those who are the focus of health, public health and social care research has been found to have a positive impact on what is researched, how research is conducted, and the impact of research findings on services and in the lives of those involved [9, 31]. By making use of people’s knowledge, lived experience, and networks, public involvement in research helps to make it more relevant and useful to the end-users and ultimately leads to better services, treatments and care [32]. There is an expectation inherent in the UK National Health Service Constitution “that patients, service users and the public participate nationally and locally in the development, implementation and accountability processes of health and social care policy and services” [11], p. 2). Statutory guidance outlines how health and care systems should build positive and enduring partnerships with people and communities in order to improve services and outcomes, including engagement, co-design and co-production [21].

The United Nations Convention on the Rights of the Child (CRC): [36] states that all children have a right to the highest possible standards of both healthcare and to have a say in matters that affect them. This can be at the level of individual decision-making (CYP’s participation in decisions that affect their own lives) and at a more strategic level (e.g., involvement in service development, evaluation and research). The CRC-informed understanding that children should be involved in decisions which affect them is increasingly reflected in law, regulation, policy, and research guidance. Further, Article 13 of the CRC states that children have the right to seek, receive, and impart information and ideas of all kinds. The realisation of children’s participation rights requires their translation into policy and practice, as well as CYP’s participation in conceptualising and realising these rights [30]. Children’s rights have been reflected in CYP’s involvement becoming an increasing priority in UK healthcare [3, 20, 38] and increasing interest in CYP’s involvement in health, public health and social care research (e.g. (e.g. [5–7, 14, 23, 26, 27, 34]). This paper is underpinned by considerations of both impact and a rights-based approach in which CYP were involved throughout the process, including the development of this article.

### Co-production

While there are debates about both definitions and practice of co-production (e.g. [2, 25, 29]) in UK health and social care research it is generally understood to be “an approach in which researchers, practitioners and the public work together, sharing power and responsibility from the start to the end of the project, including the generation of knowledge”[22], p. 1). It is something which “brings together different forms of lived or living and learnt (personal and professional) knowledge, understanding, and experience, for better outcomes and mutual benefit” [10].

From the start we sought to develop our young people's advisory group (YPAG) with a co-production ethos, while acknowledging that co-production can be particularly ethically and pragmatically complex when working with CYP [24]. Unequal power dynamics exist between adults and CYP [18], plus a young person’s advisory group by its very nature is focused on advising researcher-led projects, rather than co-producing research. In practice therefore, a ‘coproduction ethos’ for us meant seeking opportunities to co-produce as well as advise on research and working together to develop a group shared identity [12, 19].

### Children and young people’s involvement in practice

As public involvement in UK health policy tends to focus on adult input, with services for CYP seen as the ‘poor relation’ to adult services within the National Health Service [5], so the discourse on public involvement in health and social care research also tends to focus on adults [6]. Involving CYP often requires different considerations to involving adults [7] including availability (e.g. around school terms and times), consent and gatekeeping and development of age-appropriate and accessible materials and activities. The dominant model for CYP’s involvement in health and social care research in the UK is the YPAG [6], and this is the approach we chose to use when planning how to involve CYP in paediatric research at Moorfields Eye Hospital. The YPAG model allows a group of CYP to develop an understanding of research issues and processes through practical experience and training. YPAGs located within institutions, like eyeYPAG, also provide a convenient way for researchers to get input from CYP on a one-off or ongoing basis by attending YPAG meetings supported by experienced facilitators. Thomas et al. [35] discuss some of the ethical and methodological complexities in practice. For example, assumptions which can be made about CYP’s abilities to understand and make useful contributions to research, or the need for CYP to be protected from harm, which can then override their right to choose whether or not to be involved. In our experience, and the growing body of literature cited throughout this paper, it is entirely possible, with careful consideration of age-appropriate methods, for CYP to make meaningful contributions to complex research studies. This paper explores the learning from our evaluation of the eyeYPAG on how best to do this. Despite the growing evidence base (see ‘Involving young people in research’ above, Brady and Preston [7] found that reporting of CYP’s involvement in research is still patchy and inconsistent, and that more needs to be done to provide robust evidence of benefits and impacts, and the realities and challenges of CYP’s involvement in practice. We therefore wanted to evaluate our YPAG and share learning from this process, in the hope that it will be helpful to others working to involve CYP in research as well as to those interested in public involvement and engagement more generally.

The GRIPP2 checklist for reporting public involvement in research [33], this paper outlines the findings from our co-produced evaluation of the eyeYPAG. In co-authoring this paper with young people, we also bore in mind the suggestion by Scholz and Bevan [28] to go beyond the requirements of the GRIPP2 checklist and engage in reflexive research practices with public participants (in this case young co-evaluators). So, this paper was developed collaboratively with the young co-authors, who helped plan the paper and reviewed and contributed to drafts.

Young co-authors perspectives: why is it important to involve CYP in research?^1^ Jasmine: It is important, because a lot of the time it is them who are receiving treatment, and it is important for them to learn about treatments and research and to give advice.

Rhianne: It is what affects them, and ultimately the research is to help future generations, which is today’s children.

Eleri: I believe that it is important to involve young people in the research because much of the research is about young people therefore not including them can feel counterproductive. Not having young people involved could mean that the outcome of the research will be biased to the adult view. Having a diverse age range also ensures that all viewpoints are included and acted upon, and each person feels a sense of belonging and gets the recognition they deserve! For young people, learning about healthcare could change their perspective in different ways and make them more understanding of how much effort is needed!

### eyeYPAG

eyeYPAG is a YPAG for eye and vision research based at Moorfields Eye Hospital (https://generationr.org.uk/eye-ypag/). eyeYPAG is part of a wider national network (Generation R) made up of local YPAGs across the UK; the European Young Persons’ Advocacy Group (eYPAGnet; www.eypagnet.eu), a virtual consortium of YPAGs that supports the involvement of CYP in European clinical trial design and health research and the International Children’s Advisory Network (iCAN; www.icanresearch.org); a worldwide consortium of children’s advisory groups.

ADN and JM, with support from LMB, recruited CYP through their existing networks, focusing initially on CYP who had participated in research at the Children’s Eye Centre. This included CYP with eye and vision conditions and others with an interest in eye and vision research, including siblings and those who had participated in studies as ‘healthy volunteers’.

In the group’s first year (March 2019–February 2020) the group met 5 times, on a Saturday for 4–5 h including breaks, at or near the Moorfields Children’s Eye Centre with the aims of:

- Teaching CYP about research and eye conditions
- Teaching researchers about the involvement of CYP in eye research
- Giving CYP a voice in eye research
- Researchers working together with CYP to improve the design of their projects for CYP
- Working with JM specifically on her PhD project, which aims to co-design improvements for children’s eye research experiences
- Working together to ensure information about research is CYP friendly
- Sharing ideas on what should be researched and how
- Establishing a co-production ethos for the group

In the second year eyeYPAG changed to meeting online due to the COVID-19 pandemic, with meetings held on Zoom on 8 Saturday mornings between March 2020 and June 2021 (when the evaluation was conducted). We found that online meetings needed to be shorter, as it is harder to be interactive and concentrate online, so these meetings were normally about two hours long.

We wanted to evaluate the eyeYPAG so that we could learn from everyone involved what has been good about the group, what difference we have made and how we can develop and improve in the future. This article outlines how a group of eyeYPAG members co-produced the evaluation [1] with the group’s facilitator, Louca-Mai (LMB), and what we learned about the group, young people’s involvement and co-producing an evaluation of public involvement.

## Methods

### Aim, design and setting of the study

Our project was a co-produced self-reflective process evaluation [16].

Our aims (agreed at our first meeting):

- To find out what difference the eyeYPAG has made to research, and to group members
- To understand what has worked well and what could be improved after the first two years of eyeYPAG
- To use what we learn to help improve and plan for the future of the group, and help other people interested in involving CYP in research

### Participants and process

21 CYP were involved in eyeYPAG since the group started in March 2019, and at time of the evaluation the group had 14 active members aged between 10 and 18 (11 female and 3 male). 7 group members had eye and vision conditions, 3 were siblings of group members with eye and vision conditions and the remainder joined because they had taken part in clinical trials and/or have an interest in eye and vision research. All YPAG members were involved as evaluation participants through participation in focus groups and/or completion of an online survey. We also collected data from parents and carers of YPAG members, researchers who had worked with the group, our funders and the group’s facilitators (who also co-authored this paper).

Once we had developed our aims we decided on our methods and developed recruitment and data collection tools during our online meetings using Jamboard to post ideas and Googledocs to finalise the questions for the surveys and focus group ‘topic guides’, and agreed who was going to ask which questions in the focus groups. As the project took place during the COVID-19 pandemic all data collection took place online in March–May 2021 through:

- Workshops with eyeYPAG members during the group’s March and June 2021 meetings led by young evaluators with support from LMB and JM. In the March session we asked the group (4 females (aged 11,11,12,12) and 2 males (aged 11 & 13) for their views on eyeYPAG: the benefits of being in the group, what they had learnt and would like to learn, and what they liked and thought could be improved. In June the young evaluators (4 females (aged 12,12,12,17 years) and 1 male (age 11 years) presented our draft findings, and asked for feedback, so that the wider group (5 females (aged 10, 11,11,16,18 years) and 1 male (aged 13 years), 4 of whom had also attended the meeting in March) could inform our analysis and reporting.
- Survey of current YPAG members (10 responses) which looked at the same things as the March meeting above. As CYP had the option to complete the survey anonymously, to encourage honesty and participation, it is not possible to ascertain non-responses but all YPAG members were given multiple opportunities to participate.
- Focus group with researchers (n.5 of 12 who have previously worked with the group, some on multiple occasions) led by LMB with young evaluatorsasking some of the questions, as we thought they would be more honest that way. We asked researchers about what they expected before meeting the YPAG, how they found working with us, and how our involvement had made a difference to their research.
- Survey of researchers (4 responses, one of whom also participated in the focus group) which looked at the same things as the focus group above. 4 non-responses.
- Survey of parents and carers (7 responses from 9 possible participants, as the group has several siblings) exploring why they supported their child to join the group, how well the timing and location of the meetings worked for them, their perspectives on the benefits for their child of being a member of the group, and anything that could be improved.
- Focus group with the three people who help organise the group (LMB, JM & ADN). The young evaluators planned and led this session. We asked why they had set up the YPAG, what assumptions and expectations they had at the start about working with CYP, and how this changed. We also asked how successful they thought the group had been so far, what the challenges had been and how more young people could be involved in research.
- Survey of the two main funders of eyeYPAG (2 responses)

### How we worked together

We met regularly during the project on Zoom, shared information and ideas by email and worked on project documents in between meetings on Googledocs. LMB did some training sessions in these meetings on research methods and ethics and all the big decisions about the project were made together, with young evaluators deciding what they wanted, and were able, to get involved in. We kept a record of how many hours we all worked on the project so that everyone could be paid fairly for their contribution at the end (LMB was paid to work on the project as part of her job and the young evaluators were paid an hourly rate).

### Analysis

**Fig. 1:**
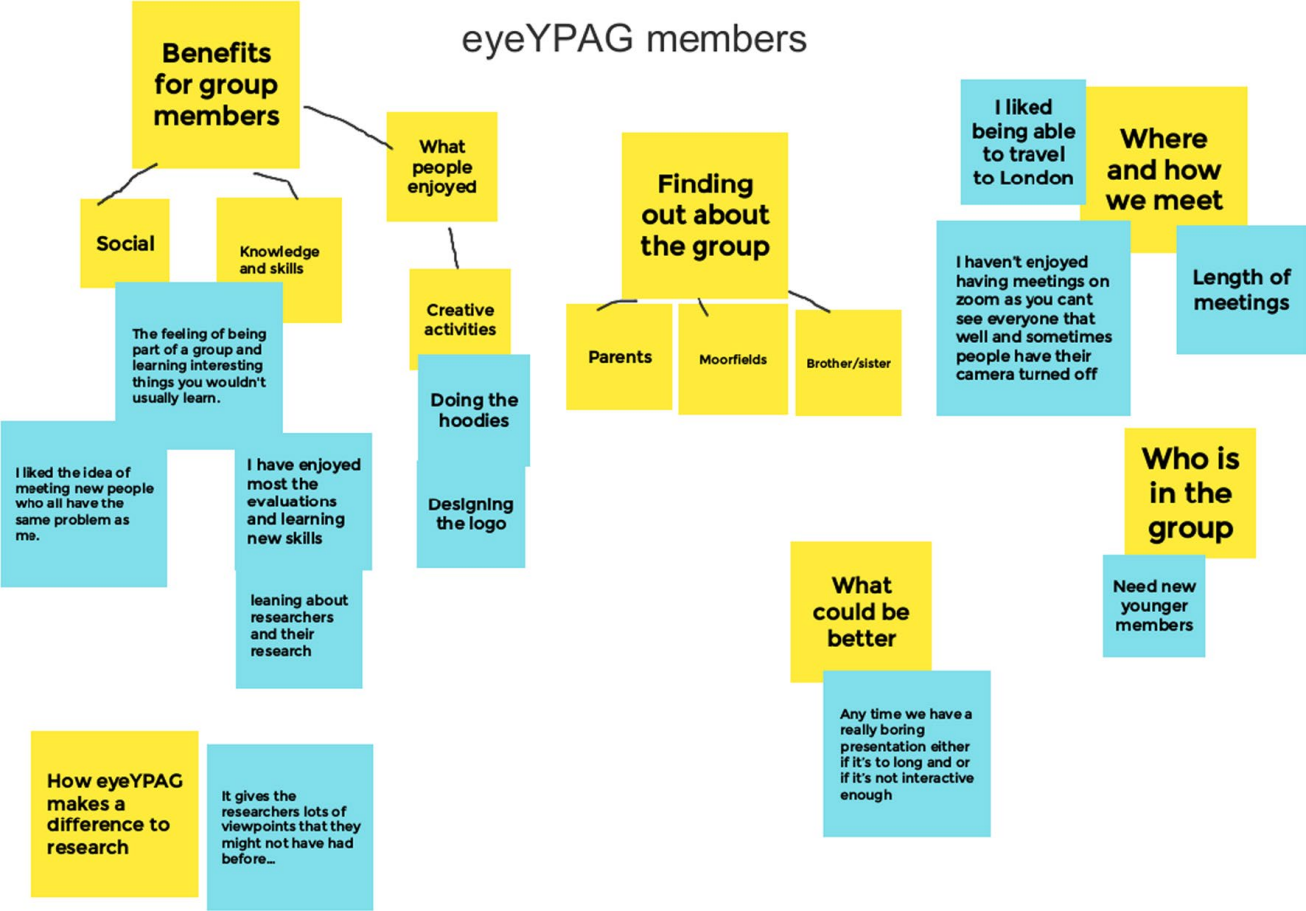
example collaborative analysis using Google Jamboard

**Fig. 2:**
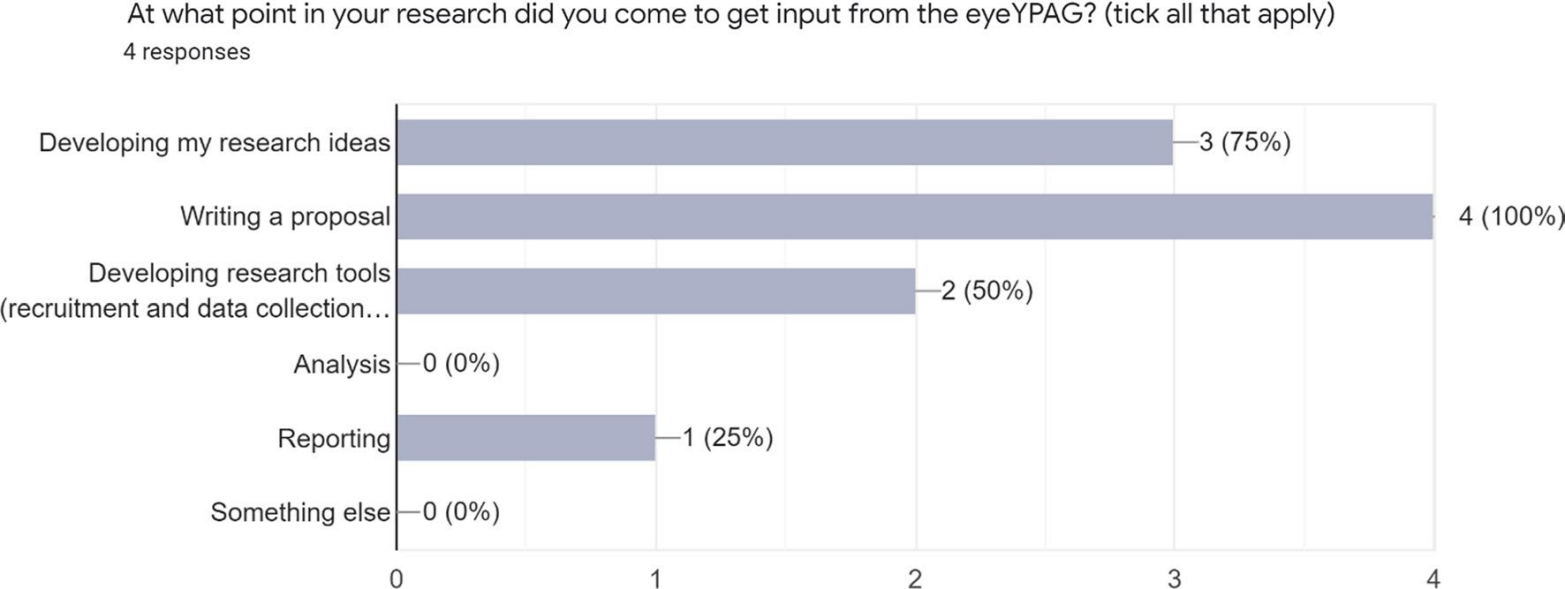
Research responses to questions on stages of research YPAG were involved

We recorded and transcribed the focus group conversations and then analysed this material and qualitative data from the online surveys using a reflexive thematic analysis approach [8]:

- Familiarisation and collaborative data coding: We read through all the data we had collected and made notes about our ideas. Then we went through the transcripts and qualitative survey answers and coded things that seemed relevant to our research questions. We did this in different ways that worked for us – e.g. by highlighting words which related to each code in a different colour in a transcript, or by creating a table to organise data related to different codes.
- Theme development and review: We got together online to decide what our initial themes would be, based on the coding we had done, grouping together codes which seemed to be related or linked (Fig. 1). We then went back to the YPAG to ask group members what they thought about the themes and codes we had identified (workshop 2 above).
- Theme refining, defining and naming: We asked LMB to do the rest of the analysis, so she put all the data in NVivo qualitative analysis software, using the coding the group had done and working with the themes we had identified. We reviewed the themes and talked about the analysis in our meetings.
- [Figure 2: Research responses to questions on stages of research YPAG were involved]

Quantitative analysis was limited by the small number of participants, and so we analysed this in Excel and reported descriptive data.

## Results

This section outlines our findings, organised by our main themes: the benefits of involvement, how the YPAG worked ‘in practice’, what is needed to support the group (facilitation) and planning for the future.

### Benefits of involvement

Benefits discussed by participants included a sense of belonging, gaining knowledge and skills (learning), making a difference (impact) and an awareness of children’s rights and voice.

### Belonging

YPAG members, in the survey and focus groups, and parents and carers who responded to the survey. talked about joining the group in order to meet other CYP with similar interests or experiences, or finding this a benefit of being a member. During the focus group facilitated by the young evaluators facilitators also reflected on how the group had bonded really well. YPAG members talked about valuing how we work together, reflecting the work we have done as a group on our ‘shared identity’, as well as advising on research projects:

*“[I like] meeting other young people who are also passionate about getting their voices heard and doing it in a way that we can all work together in a family-like way.”* (YPAG member)

*“Being able to identify as part of a group, where she has a voice, has been good for [my daughter]. It has also helped her to identify as someone who has a visual impairment and to recognise that she is not alone in this.”* (parent)

Group members also valued the wide age range of the group, as did facilitators:

*“I think some groups I work with, you end up splitting up the older and younger members of the group more. Actually, with this group… the older group members help the younger ones. So I think it’s worked really well.*” (facilitator)

### Learning

YPAG members members valued learning new things:

*“I get to learn about different eye conditions and help take the research forward to help people with the conditions.”* (YPAG member)

Knowledge and skills which parents and carers said in the survey that their children had gained included:

- Understanding what problems children with eye/vision conditions face
- Appreciating how CYP can help adults design more meaningful and realistic research
- Learning to critically appraise other people's work and ideas
- Giving them the confidence to take on additional work and responsibilities (if and when they wanted to do so)
- Developing skills working with adults and children they do not know
- More confident about sharing their views
- Participating in a process with tangible outcomes
- Experience of working with others (adults and CYP of different ages) and independently

Facilitators in the focus group reflected on both the value of sharing knowledge and teaching CYP and, conversely, how much they had learnt from YPAG members, both in terms of their perspectives on research and how to pitch their involvement:

*“So from the very first meeting I've been so humbled really, by the insights and how well children and young people can talk about their experience”* (facilitator)

*“I have learnt so much from the children and young people. That for me, was the biggest learning experience here.”* (facilitator)

### Impact: young people’s perspectives

In the focus groups YPAG members discussed the importance of feeling that they were making a difference to research:

*“[The researchers need young people’s] point of view… because we're the ones they're researching [so] we have our say, and it helps them, it helps us, it helps everyone.”* (YPAG member)

*“[Researchers who come to the group] will build on the idea you've given… if you give an idea, they'll tell you maybe, why they can't do it, or suggest a better alternative using your idea or they'll just say ‘yes, that's a really good idea’*” (YPAG member)

In terms of impact on CYP of being group members, in the survey CYP (n = 10) were asked to rate their experience of the group in relation to the following statements (Table 1):

**Table 1:** YPAG members responses to survey questions on experience of being a group member

During eyeYPAG meetings I feel that…	Always	Mostly	Sometimes	Never
My views are listened to	8	2	0	0
Things are explained in ways that I can understand	10	0	0	0
I’m able to ask questions	10	0	0	0
I can get involved in decisions about the group and how we work together	7	3	0	0
Information given before and during meetings is accessible to me	8	2	0	0

In open text responses, YPAG members said that things the group did well include:

- *“They allow everyone to have a voice and make everything accessible to everyone.”*
- *“It’s a fun, interactive and very interesting group.”*
- *“Even in lockdown, we've still been pretty good at working together and getting to know each other better, so it's not as lonely, and we know that our voices are valued more, and that people are actually going to listen.”*

### Impact: adult perspectives

All researchers who took part in the focus group or completed the survey reported some impact from the YPAG’s input into their research. This included changes to methodology, research protocols, recruitment and data collection tools:

*“We have taken some concrete suggestions on board, and made tweaks to tests… It also provided helpful supporting material for at least one grant application and one peer reviewed paper”* (researcher)

*“There were things that the group told us they wanted to change I had never thought about… It really opened my eyes and gave me a different perspective… it was incredibly helpful.”* (researcher)

*“[Involvement] makes it much more suitable for the children and young people who take part in the study … [and] that then just makes it more successful in the end, either in the numbers of people willing to take part, because it's an appropriate project for children, or having a better experience while they're taking part.”* (researcher)

*“I think it's actually changed, not the research, but me as well… learning what children or young people can say, how much they can give to research, has actually set me up quite early on in my career to make sure that I continue to [work with young people and] make the research better.”* (researcher)

The feedback from researchers about coming to eyeYPAG meetings was overwhelmingly positive, with comments about ‘invaluable advice’, ‘insightful comments and suggestions’ and ‘excellent engagement’. This was echoed by funders in their survey responses:

*“Children and young people can have very different perspectives and expectations of research and it is critical their voice is heard. They can have great insights that adults will fail to spot or think less important, but which might make all the difference when engaging their peers in research studies. However, their involvement is not only important in the design of research but also for the future of research as they’re helping to shape the research (and researchers) of the future*.” (funder)

They went on to consider success in terms of impact to research, as described above, and how, from their perspective, they had achieved their aims and aspirations when setting up the group regarding teaching, giving CYP a voice, and improving and informing research. Facilitators reflected in their focus group on what they considered the a success of developing a group informed by co-production principles:starting with open minds and high aspirations, but acknowledging that with such a young cohort (some aged 8–9 years old at the start) with little prior experience of research or involvement, that an approach of ‘working towards’ co-productionwas required.

*“They didn't have, a lot of… experience of being involved in something like this before the group started. So we had to work up towards that.” *(facilitator)

Facilitators discussed the group’s impact in terms of dissemination:

*“It's not just changing eye research. We've done things like the podcast and things like that, we've spoken at conferences,…. a few of us [facilitators and group members] went to a Co-production Collective event and talked about our experience and what we've learnt about having meetings online. There's lots of interest in the group outside of just eye and vision research.”* (facilitator)

### Children’s voice and rights

As discussed in ‘Background’, CYP have a right to be involved in things that affect them, including research. Participants from all cohorts mentioned this.

*“I wanted to help young people get their voices heard, especially in a healthcare environment. I thought that I could make a difference.”* (YPAG member)

*“It's incredibly unethical not to allow [young] people to have a voice in how research that involves them is being undertaken.”* (researcher)

*“In healthcare, we always have to stand up for children and young people, and I feel that has to do with them not having a voice and their parents not having a very loud voice. Whereas, other patient groups are bigger and more articulate.”* (facilitator)

### YPAGs in practice

When discussing the reality of involvement adults talked about the importance of planning and support, and of considering accessibility, particularly when working with a group which included CYP with eye and vision conditions. Participants highlighted the importance of allowing time and space for creativity and how the COVID-19 pandemic had created opportunities to do involvement differently, as well as presenting challenges.

### Involvement in different stages

Participating researchers said that they came to work with eyeYPAG because they already knew about the group or had been told about the group by someone who knew them. Many came to the group in the planning stages of their research:

On-going involvement was encouraged, and several researchers worked with the group more than once and were keen to come back to the group later on in their projects. Two of the facilitators and co-authors of this paper (JM and ADN), also work with the group as researchers:

*“[eyeYPAG are] my advisory group for my PhD.. [so] I’ve been working with the group since the beginning. I've had about four sessions with them exploring different parts of my project, and they've just been totally brilliant, helping me improve it*.”.

### Planning and support

Researchers and facilitators highlighted the importance of thoroughly planning sessions before coming to meetings.

*“Try and make it as easy to understand as possible… break it down… and really just focus on one single element, because it's quite difficult to cover lots of things at once. It takes time to explain, make it fun, and give enough time for everyone to express their views on what you're saying and input into it.”* (researcher)

Researchers valued the support they had had from the group facilitators to do this.

*“[The facilitators] have so much knowledge, and they are able to provide their suggestions in a constructive, collaborative way.”* (researcher)

### Accessibility

Researchers also valued the personal and practical experience of the group of having, or knowing about, eye and vision conditions and research. In a group with a range of visual impairments and other access needs accessibility was an important planning consideration:

*“A challenge that you need to overcome for this group is the accessibility of the information that you're presenting. Even harder online, because you have no idea what device people are using or what their internet's like… and, most importantly, how much they can see. You really have to think ‘how can I make this accessible to everyone?’*” (researcher)

### Space for creativity

Alongside working with researchers, an aspect of eyeYPAG meetings which group members really valued was creative activity:

*“I find it easier to learn things and also feel like my voice is being listened to when we do things creatively instead of just sitting there.”* (YPAG member)

**Fig. 3:**
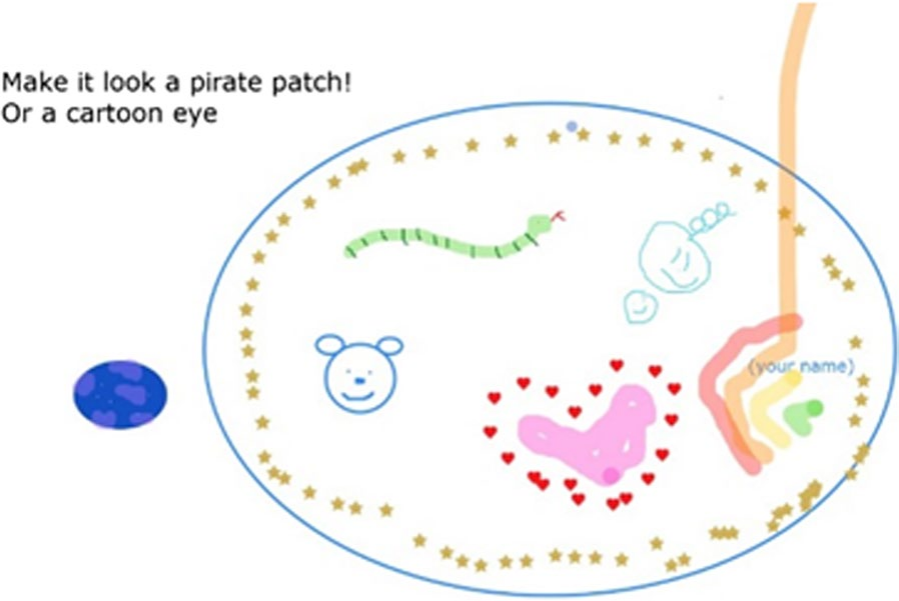
Screenshot of Zoom whiteboard from online meeting

Creativity was also valued by researchers:

*“We had an activity where we all drew what we think eyepatches should look like [using Zoom whiteboard in an online meeting, see Figure **3**], and I felt that that was really good at helping the young people to contribute what they thought… the creativity was something that I've not seen before and was really impressed with.”*

### Top tips

‘Top tips” from researchers and facilitators for planning a YPAG meeting:

- Think carefully about exactly what you want to get from the session and how to make best use of the time
- Present a very small part of the project and explore in depth
- Work with the facilitator beforehand and plan the session well to make it interactive and engaging
- Don’t just do a presentation and question and answer session: “Consider activities for children with all needs, to ensure maximum participation”
- Keep your presentation short so you have lots of time to talk about it
- Make sure you explain your ideas/technical terms clearly and in a way that young people will understand
- Be prepared to make some changes to your project

### COVID-19

The need to move YPAG meetings online during the COVID-19 pandemic presented challenges including accessibility, keeping members engaged and the need to adapt quickly to new ways of working:

“*When we met at Moorfields we used to make our meetings really creative, because that helps engage everybody and be interactive… Then COVID took that option away. So I felt quite nervous about how we were going to manage to keep people engaged… [but] we made some real progress with making things interactive and creative in online meetings too.”* (facilitator)

*“it was a bit nerve-wracking moving online and … [making] sessions accessible to everyone in this format.”* (facilitator)

The solution was to reflect together on how best to make online meetings as engaging and accessible as possible, as it was a learning process for all of us. Our members co-authored a blog post and presented at a UCL Centre for Co-production network meeting on our learning [12]. While many group members said that they missed the social and fun aspects of meeting in person, and that online meetings could be less interactive and engaging, the preference of most group members and adults going forward, was for a mix of online and in person meetings.

Young co-authors perspectives: lessons from the evaluation on how best to involve CYP in research Jasmine: You need to capture children’s attention and be aware that they may understand a lot more than the researchers think, they may just need less complicated words.

Rhianne: Don’t assume that children are unable to understand—don’t speak to the parents only. Explain it to the children, because the parents often cannot explain it as well as the doctors and researchers.

Eleri: From the evaluation we learnt that you really must have a range of inputs. We realised that without young voices the research may not end up positively affecting young people enough. In addition, it is amazing opportunity for young people to get involved with. Along with meeting new people, young people learn so much about the healthcare system and get an insight into how it is run. Considering many people in YPAG have eye issues, it is wonderful for them to know how much work goes into ensuring eye healthcare is safe, reliable and incredible altogether!

### Behind the scenes: facilitating a YPAG

As discussed above, support and facilitation was key to the success of the group for both young people and researchers.

**Enabling factors** included the role facilitation played in supporting researchers:

*“Because we'd had a rehearsal [with the facilitator], it probably went better than it could have done, but even when you do rehearse what you're going to say to children and young people… there are always things that you assume would be really obvious… and you're seeing the blank faces, you're thinking ‘oh we could really have explained that better’.”* (researcher)

Researchers also reflected on the shared agreements film co-produced by the group [19] which they found a really helpful way to understand eyeYPAG’s approach.

Creative and social activities were also a key element of successful facilitation, as discussed above, both to build group identity and make the involvement process more engaging and fun. Funding for tablets also helped make documents more accessible for group members during in-person meetings, as well as sending documents out in advance for online meetings so that they could be printed or put on a device at home if needed:

*“We made some real progress with the face-to-face meetings making things accessible. We got the iPads, we were really getting to know each other and doing the social things, went out for lunch and all that sort of thing.”* (facilitator)

**Challenges** included resourcing and administration:

“*The administration side, the really boring side has turned into a challenge that I hadn't anticipated, but of course, having the group is just completely worth it and going through all the hoops.”* (facilitator)

While the researchers who took part in the focus group and survey were enthusiastic and engaged, others had taken more convincing:

*“Some researchers are really keen and some researchers you have to do a bit of work on to convince them to give up their Saturday morning and that it's actually going to make their research better.”* (facilitator)

*“I think with eyes and vision [research] there are so many sub-specialities….there's an impression that children maybe have one condition, wouldn't be about to speak another condition…. [but] children can definitely give a value to projects regardless of whether they have that specific condition, and we need to really bring that home as well*.” (facilitator)

### Future direction

After two years group members and facilitators were of the view that a refresh of the group was needed, as some of the original group members had left or would do so in the next few years as they began to move on and ‘age out’. It was felt that this needed a good application process to both involve CYP with a range of interests and experience, and to get an understanding of interests and access needs. More engagement was also needed within the hospital:

*“I don't think as many families [coming to the Children’s Eye Centre] as we would like know that there are research opportunities. So we can do some in-house advertising as well.” *(facilitator)

It was felt important to involve current members in developing a recruitment strategy and materials. In addition, the facilitators discussed the need to think beyond the YPAG model and consider other ways to involve CYP who may not be interested in, or able to, have ongoing involvement or come to meetings in a hospital (e.g., through working with schools or services). But thought was also needed about how to integrate new members into the group, and how existing group members could support or mentor new ones.

Finally, another important challenge for facilitation is staffing. The lead facilitator (LMB) left for a new role soon after this evaluation was completed, and for almost a year afterwards there was nobody with dedicated time to take over this role, meaning that other facilitators had to manage this in addition to their regular jobs. So often YPAGs, and public involvement more generally, rely on one or two people who take on the role because it is something they care about with little or no funded time to do this and/or a lack of job security. When people leave this can then create loss of knowledge and continuity.

## Discussion

### Learning from the evaluation

We wanted to evaluate eyeYPAG so that we could learn from everyone involved what has been good about the group, what difference we have made and how we can develop and improve in the future. We found that the eyeYPAG has made a difference to research and to group members. We learnt that while we aspired to, and largely achieved, a ‘coproduction ethos’ for the group, there is still work to be done. Whilst it was great that we had no ‘sometimes’ or ‘never’ answers (see Table 1), we will continue to focus on making sure the answers are ‘mostly’ or ‘always’ for everyone.

**Table:** 

*Recommendations we made at the end of our evaluation report*:	
Resume in-person meetings alongside some online meetings	
More creative sessions	
Make sure all research sessions are engaging, accessible and interactive	
Refresh the group membership	
Develop guidance/top tips for researchers coming to meetings	
Support group members to set personal goals (e.g. write a blog post, give a presentation) and make a plan for how to achieve these	
Continue to focus on and develop our ‘coproduction ethos’	
Let more researchers know about the group (e.g. make a promotional film)	
Make sure we continue to evaluate and collect information on eyeYPAG and the difference it makes to research	
Ask researchers to credit the group and, if possible, include them in publications	
Think about the long-term future of the group, including getting included in more funding bids	

### Young co-authors’ perspectives: being a young evaluator

Jasmine: I have learnt that is very hard to get people’s honest opinion. There is a need to be respectful, but still encourage people to express their view. I benefited from widening my skills in this evaluation project, like interviewing skills, framing good questions to ask, and asking open questions. It would have been even better if we could have used more creative methods as well as standard surveys.

Rhianne: I learnt about managing a project, from planning to interviewing, making surveys, getting information out of the transcript. I benefited by getting a deeper understanding of research and by hearing other people’s ideas and opinions. I would prefer to meet face-to-face for a longer session—having several online sessions took a long time, and I felt it was difficult to keep track, because school is so busy.

Eleri: I am so grateful that I was given the opportunity to be a young evaluator because I know that not everyone in is handed such a rewarding opportunity. Being someone who has dealt with eye issues from birth, it was extremely enlightening to learn about the process behind it all. Being a young evaluator meant that I learnt so much about eye research around the world but also local which for me was a very humbling experience. I have gained many life skills and met many amazing and influential people! If I had to change anything I think I would included more face to face meetings but I understand that it was hard due to the world only just starting to recover from COVID-19.

### Implications of the findings

While CYP’s involvement in UK health research is an area of growing interest, they are still often the ‘poor relation’ in both health services and research [5]. The reporting of CYP’s involvement in research is patchy and inconsistent, and more needs to be done to provide consistent and comparable evidence of these benefits and impacts, and the realities and challenges of involvement in practice [7]. Our evaluation highlights important learning regarding both the process and impacts of involvement (how CYP are involved and what difference this makes). Impacts include benefits for research such as improved proposals, plans and recruitment and data collection tools; as well as YPAG members, and the adults who worked with them, gaining valuable skills and experience. But other, less tangible, benefits were equally valued including a sense of belonging and the group being an inclusive and supportive space. Meaningful involvement is not just about CYP providing advice and support for researchers, though this is of course important, but equally so is the need to do this in ways that work for CYP, embedding accessibility, creativity and building group identity.

As discussed in the introduction, it is entirely possible, with careful consideration of age-appropriate methods, for CYP to make meaningful contributions to complex research studies. The challenge is not that the research is ‘too difficult’ for CYP to understand, but that the research materials and associated activities, explanations and format are  not sufficiently clear, accessible or succinct. A key role of the YPAG facilitator is to support this process as a mediator or translator: working with researchers to ensure that their material is age-appropriate and sufficiently engaging, alongside supporting CYP to ensure that their input is relevant and useful for the research.

Skilled facilitation, and time and resources to do this well were identified as key to the success of the group. This echoes an emerging literature on the importance of facilitation in effective public involvement, including as a catalyst for discussion, reflection and relationship building [15] and in the process of providing feedback [17]. But investment in facilitation, as well as recognition of the skills and expertise involved, is also essential [37]. The fact that the YPAG included both young people with eye and vision conditions as well as siblings and participants without eye and vision conditions brought a range of diverse perspectives which YPAG members, as well as researchers, really valued. But this again needed skilled facilitation to manage as well as establishing clear ground rules about valuing different perspectives and experiences.

From the start we sought to develop eyeYPAG with a co-production ethos but, echoing Pavarini et al. [24] found that the reality of co-producing with CYP can be complex. A young person’s advisory group by its very nature is focused on advising projects led by adults, alongside the challenges of co-producing something with children (some as young as 8 at the start of the project) who had no prior knowledge or experience of research, public involvement or co-production. We were aware that  what works best for one child, young person or group does not necessarily work for all and, with the aim of actively ‘working towards co-production’, from the outset discussed and reviewed with the YPAG when, where and for how long we met as well as what we did in the meetings. We identified opportunities to co-produce as well as advise on research, evolving the way the group worked together and developing their shared identity [12, 19]. Co-producing the evaluation also involved some shared learning, particularly as the COVID-19 pandemic meant that all the work was done online. While convenient in many ways, this meant that most of the work happened in meetings, with some work in between using online file-sharing. The young co-evaluators were often busy or unavailable in between and delegated a lot of the day-to-day work on the project to LMB, particularly in the analysis and reporting stages. But, as with authoring this article, keeping the co-production values of sharing power and responsibility at the centre of our approach, does not mean everyone doing everything. The young co-researchers were given opportunities to be involved at every stage, in ways that worked for them. But it is important to acknowledge the realities of young people’s lives, and educational and other commitments mean that their capacity and availability is often limited and can fluctuate. For co-production to be feasible in reality this needs to be acknowledged, with opportunities for involvement to fluctuate, echoing the idea of ‘pockets of participation’ [13]. Similarly, taking a report co-authored by young people and aimed at a young, as well as professional, audience and developing it to fit the conventions and expectations of an academic journal also requires a balance of effort and negotiation of roles and responsibilities. In this case the lead and second author carried out much of the drafting following some initial planning meetings with all authors, with other credited authors involved in commenting on drafts and contributing the material in text boxes.

YPAGs provide a convenient way for researchers to get input from CYP on a one-off or ongoing basis but they are not, as discussed in the introduction, the only way to involve CYP in research [6]. Indeed, there is an argument for more inclusive approaches in order to involve CYP who are marginalised and less often heard [4]. While not a key focus of this evaluation, the project did highlight the possibility of considering other ways to ‘reach out’ to diverse CYP, for example through engaging with schools, youth services, patient groups and organisations working with CYP (e.g., visual impairment and disability charities) alongside a ‘core’ YPAG. So, for example involving CYP with lived experience of particular conditions or treatments in relevant studies. Some of these CYP  may then want to go on and join the YPAG, but some may prefer one-off or limited involvement. The key principle is to evolve the YPAG in collaboration, and if possible co-produced, with both existing YPAG members with others who may be interested in becoming involved.

### Limitations of the study

This was a small study with a limited and self-selecting sample; staff and CYP’s capacity also limited the scope of the project. YPAG members were sent several reminder emails and messages, as well as being reminded about the evaluation survey in meetings. But it was more difficult than expected to get all group members to take part in the survey, as we were all working remotely due to the COVID-19 pandemic, and we were doing the research during exam time. It is not possible to make wider generalisations about CYP’s involvement in health research, but we hope that it provides some useful lessons and areas for further study, as well as providing a worked example about how young people can be involved in evaluating their involvement in research.

## Conclusions

This evaluation builds on the growing evidence base on CYP’s in UK health and social care research outlined in Background, not least by involving. them in the development of that evidence, including co-authoring this article. In our view any article on CYP’s involvement is incomplete without their perspectives.

Establishing the group with an awareness of co-production principles and a children’s rights-based approach was essential to starting to shift the balance of power and challenge traditional ways of working. There were no experts—we all brought knowledge and expertise/experience but focused on learning together, not least during the COVID-19 pandemic when we had to rapidly develop new ways of working together.

While the focus of this paper is on the involvement of CYP in health research in the UK, Many of the issues raised are relevant to the involvement of adults as well as CYP, and to organisations beyond the UK supporting public involvement in research. Support and facilitation were key to the success of the group for both CYP and researchers and this is an area that needs further research. Further research is also needed on how, when and where CYP are involved in health research, how involvement can draw on other approaches and disciplines to develop more inclusive and diverse approaches, and the ethical issues raised by involving (or not involving) CYP in research.

## Data Availability

The datasets generated and/or analysed during the current study are not publicly available due to risks of compromising confidentiality and individual privacy, particularly regarding child participants. However further information data is available in the published report [1] and data is available from the authors upon reasonable request and with permission of the NIHR Moorfields Biomedical Research Centre.

## Abbreviations

CYP: Children and young people
PPI: Patient and public involvement
YPAG: Young people’s (research) advisory group

## Footnotes

1 We wanted youth voice to be heard directly in this paper, but the views of co-authors are distinct from the participants quoted in results, as the co-authors are commenting from the perspective of being a young evaluator rather than as research participants.
